# Adverse Events in Patients with Inflammatory Bowel Disease Treated with Advanced Therapies: A Nationwide, Population-Based, Propensity-Matched Cohort Study

**DOI:** 10.3390/jcm15072562

**Published:** 2026-03-27

**Authors:** Hyoung Il Choi, Jung Rock Moon, Seon Hwa Lee, Jae Myung Cha

**Affiliations:** 1 Division of Gastroenterology, Department of Internal Medicine, Kyung Hee University Hospital at Gangdong, School of Medicine, Kyung Hee University, Seoul 05278, Republic of Korea; fullhouse11245@naver.com (H.I.C.); ukino816@gmail.com (J.R.M.); 2 Center for Trend Monitoring-Risk Modeling, Samsung Medical Center, School of Medicine, Sungkyunkwan University, Seoul 06351, Republic of Korea; sproutlife@naver.com

**Keywords:** inflammatory bowel disease, advanced therapy, biologics, tuberculosis, herpes zoster, anxiety, depression, population-based study

## Abstract

**Background:** Safety profiles of advanced therapies (ATs) for inflammatory bowel disease (IBD) may be underestimated in clinical trials due to low event rates and heterogeneous adverse events (AEs). **Methods**: Using Korean nationwide claims data (2012–2022), incident IBD patients were identified and classified into AT and non-AT groups. After:1 propensity score matching, risks of tuberculosis (TB), herpes zoster (HZ), and bowel malignancy as primary AEs, and anxiety/depression as secondary AEs, were evaluated. **Results:** After matching, 11,205 patients were included in each group. Overall AT use was not associated with a significant increase in TB risk compared with non-AT therapy (hazard ratio [HR] 1.2; *p* = 0.335), whereas anti–TNF biologics showed higher TB risk (HR 2.0; incidence rate [IR] 100.2 vs. 65.4 per 10^5^ person year [PY]; *p* < 0.001). AT use was associated with a modestly increased risk of HZ (HR 1.4; IR 2165.8 vs. 2151.3 per 10^5^ PY; *p* < 0.001), with the highest incidence in small-molecule agents (HR 2.0; IR 4134.2 per 10^5^ PY). The risk of bowel malignancy did not differ between AT and non-AT groups (HR 1.2; *p* = 0.475). Both anti–TNF and non–anti-TNF biologics were associated with reduced risk of anxiety/depression compared with non-AT therapy (both HR 0.8; *p* < 0.001 and *p* = 0.004, respectively). **Conclusions:** Overall AT use was not associated with an increased risk of TB, whereas anti–TNF biologics were associated with a higher risk of TB. AT use was also associated with an increased risk of HZ, particularly with small-molecule agents, and a lower risk of anxiety/depression.

## 1. Introduction

The incidence and prevalence of inflammatory bowel disease (IBD), including ulcerative colitis (UC) and Crohn’s disease (CD), are rising steadily in Asian countries [1]. Compared with Western populations, Asian patients with IBD exhibit distinct epidemiological and phenotypic features [2]. Accordingly, adverse events (AEs) related to IBD treatment may also differ between Asian and Western patients. Although numerous clinical trials have reported AEs associated with advanced therapies (ATs) for IBD [3], real-world evidence remains essential. Randomized controlled trials and open-label extension trials may underestimate the risk of AEs due to selective inclusion and limited event rates. Thus, nationwide population-based data are needed to better characterize the safety profiles of ATs in clinical practice.

Various ATs are currently available for IBD management. Despite the growing number of options, there remains a paucity of real-world data on safety profiles, particularly in Asian populations. Given the heterogeneous and long-term nature of AT-related AEs [4], large-scale, population-based studies are needed to better understand their risks. To date, most real-world studies on AEs in patients with IBD have primarily focused on incident tuberculosis (TB) associated with anti-tumor necrosis factor (anti-TNF) therapy [5]. However, other clinically important AEs—including herpes zoster (HZ), malignancies and anxiety/depression—also warrant investigation. A recent network meta-analysis in UC patients found no significant differences in total or serious AEs between ATs [6], but such analyses are inherently limited by study heterogeneity and the indirect nature of comparison.

Therefore, we aimed to compare the risk of various potential AEs in patients with IBD treated with ATs and those treated with non-ATs using a nationwide population-based cohort.

## 2. Methods

### 2.1. Database

In South Korea, the National Health Insurance System (NHIS) provides universal coverage for both inpatient and outpatient medical services for approximately 52 million Koreans [7]. The Health Insurance and Review Agency (HIRA) reviews the reimbursement decisions on insurance claims, maintaining the adjusted medical and pharmacy claims data for the entire Korean population [8,9,10]. De-identified sociodemographic characteristics, health-care service information (e.g., diagnoses, procedures, and prescriptions), principal diagnoses, and comorbid diseases are available in the HIRA database [8,9,10], which records diagnoses based on the Korean Classification of Disease. This is a modified version of the International Classification of Disease, 10th revision (ICD-10), adapted for use within the Korean healthcare system.

The NHIS registry program launched the ‘Rare Intractable Disease (RID)’ registration program based on strict and uniform diagnostic criteria [9,10]. Since 2009, patients with IBD who are registered in the RID program are eligible for a co-payment reduction in medical costs of 10% after their diagnosis [9,10]. To be included in the RID program, the diagnosis of IBD must be based on the pre-defined diagnostic criteria provided by the NHIS and reviewed by the corresponding physicians and medical institutions. If patients are confirmed to have IBD, they are registered using a unique claims code (for example, V130 for CD and V131 for UC). Thus, the NHIS claims data for IBD are verified and reliable.

### 2.2. Study Design

This nationwide population-based study included all adult (≥17 years) patients with IBD in the 2010–2022 HIRA database. The age threshold for adult patients was based on the age classification used in the Montreal classification of IBD. Using claims data from the RID and NHIS programs, we enrolled all patients diagnosed with IBD; the codes K51 and V131 indicated UC, and K50 and V130 indicated CD. To be assigned a V code, certain strict criteria must be met [11,12]. Patients who were previously diagnosed using IBD codes between January 2010 and December 2011 (screening period) were excluded. An incident case of IBD was defined as a patient who was newly registered between January 2012 and December 2022 (study period) with no IBD claims data during the screening period. Accordingly, a 2-year lookback period (January 2010 to December 2011) was applied to ensure no prior claims for IBD before cohort entry, thereby defining incident cases during the study period. Patients who received IBD-related medications for fewer than 90 days were also excluded from the IBD cohort, regardless of diagnosis.

Incident AEs were compared between patients receiving ATs and those receiving non-ATs following 1:1 propensity score matching (PSM) based on age and type of IBD. An incident AE was defined as a newly registered event during the study period, with no prior claims related to that AE during the baseline screening period. In this study, AEs included TB, HZ, and malignancy of bowel as primary AEs, and anxiety and/or depression as secondary AEs. To ascertain the temporal relationship between IBD medications and AEs, patients with AEs that had been diagnosed before treatment initiation were excluded from the analysis. Therefore, patients were assigned to the AT cohort starting from the date of AT initiation, and only incident AEs occurring after AT initiation were included. Patients who did not receive AT were followed from the date of IBD diagnosis, and AEs occurring prior to conventional treatment initiation were excluded. The follow-up period was defined as the time from IBD registration to the date of the AEs or last date of follow-up. Patients without newly developed AEs were censored on the last day of follow-up or date of death. However, because follow-up was initiated at different time points in the two cohorts (treatment initiation in the AT group vs. diagnosis in the non-AT group), this asymmetry may introduce a risk of immortal time bias that cannot be fully mitigated by excluding pre-treatment events or by applying Cox proportional hazards models. Accordingly, this study design inherently limits the ability to achieve a fully unbiased comparison between groups.

Comorbidities were defined using ICD-10 codes and included diabetes mellitus (DM; E11–14), hypertension (I10–13 and I15), cerebrovascular diseases (I63–64), chronic obstructive pulmonary disease (J41–44), renal disease (N18–19, Z49, Z94.0, and Z99.2), and cardiovascular disease, including congestive heart failure (I50) and ischemic heart disease (I20–25) (Supplementary Table S1) [13]. Medical cost was defined as direct healthcare cost, including inpatient and outpatient expenditures associated with IBD-related claims [10]. Hospitalization was defined as hospitalization of more than 3 days, occurring via the emergency room (ER) with a primary diagnosis of IBD [14]. Surgery for IBD was defined as bowel resection surgery as described in the Current Procedural Terminology system [15]. These included small and large bowel surgeries, claimed with a primary diagnosis of IBD (codes Q2650, Q2651, Q2671, Q2672, Q2679, Q2680, Q2691, and Q2921–Q2926) (Supplementary Table S1). Death was defined as the absence of claims data and loss of eligibility from the NHIS database, which is a compulsory insurance that must be subscribed to when alive. The study protocol was reviewed and approved by the Institutional Review Board of Kyung Hee University Hospital at Gangdong (KHNMC IRB No. 2024-05-011).

### 2.3. IBD Medications

Non-ATs were categorized as 5-aminosalicylic acids (including sulfasalazine and mesalazine), corticosteroids (including topical and systemic forms), and immunomodulators (including azathioprine, mercaptopurine, and methotrexate). Topical corticosteroids in the form of eye drops, ointments, creams, gels, and lotions were excluded. Overall, ATs included anti-TNF biologics (including infliximab, adalimumab, and golimumab), non-anti-TNF biologics (including vedolizumab and ustekinumab), or small-molecule agents (only tofacitinib). Other small-molecule agents, such as filgotinib and upadacitinib, were not included because they were not available during the study period.

**Figure 1:**
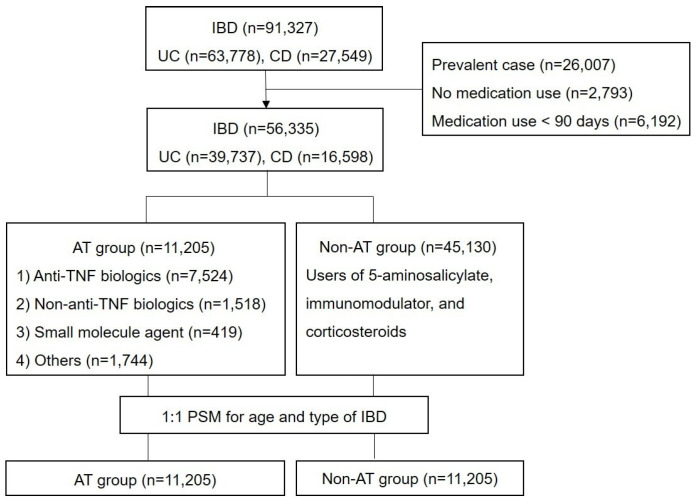
Flow chart of the study population. Among 56,335 patients with IBD, 11,205 patients received ATs and 45,130 patients received non-ATs. Among patients in the AT group, 7524 patients were treated with anti-TNF biologics only, 1518 patients with non-anti-TNF biologics only, 419 with small molecule agents only, and 1744 patients were classified as the other subgroup (those received ATs from more than one class). After 1:1 propensity score matching, same number of the AT and non-AT groups (*n* = 11,205 each) were finally analyzed. AT, advanced therapy; CD, Crohn’s disease; IBD, inflammatory bowel disease; PSM, propensity score matching; TNF, tumor necrosis factor; UC, ulcerative colitis.

ATs in Korea cannot be used as a first-line or “top-down” strategy under the NHIS reimbursement criteria. Patients must first receive conventional therapy such as corticosteroids and immunomodulators before becoming eligible for AT, and therefore AT initiation in this study almost invariably occurred after prior exposure to these therapies. In this study, overall AT group included patients who switched between different classes of AT; however, the concomitant use of two or more ATs is not permitted according to the reimbursement policy of the NHIS. The AT subgroups were classified as anti-TNF biologics, non-anti-TNF biologics, small-molecule agent subgroups or others (Figure 1). When patients received ATs from more than one class were classified into the “others” subgroup. Anti-TNF biologics, non-anti-TNF biologics, and small-molecule agent subgroup analysis were restricted to patients receiving monotherapy with a single class of AT. This approach was adopted because, when multiple AT classes are used sequentially, it becomes difficult to attribute safety outcomes to a specific AT class or to quantify the relative contribution of each therapy.

### 2.4. Definitions of AEs

(1) Active TB

Since 2011, Korean guidelines for the use of ATs (especially anti-TNF biologics and small molecules) recommend screening for active and latent TB before prescribing ATs. Therefore, all patients with IBD treated with ATs undergo routine screening for TB using a combination of patient history, chest radiography, and the interferon-gamma releasing assay and/or tuberculin skin test before AT initiation [5].

In the NHIS, reporting incident TB cases is mandatory according to TB-control legislation [16]. All patients with TB are registered in the HIRA database using the ICD-10 codes A15–A19 or U88 (Supplementary Table S1) or the RID registration codes V206, V246, or V231. After registration in the RID system, patients were reimbursed for the medical costs associated with TB treatment. Patients with active TB were defined as those with corresponding diagnostic codes and prescriptions for ≥2 anti-TB medications for a minimum duration of 4 months, to exclude potential cases of latent TB infection (LTBI).

To distinguish between patients with LTBI and those with active TB, LTBI cases are registered using an R76.80 or Z22.7 ICD-10 code (R76.80 was used until 2020 and Z22.7 from 2021) and a V010 RID registration code. The current Korean guidelines for the treatment of LTBI recommend daily isoniazid monotherapy for 6–9 months, daily rifampin monotherapy for 4 months, or daily isoniazid and rifampin combination for 3 months [17]. Patients were excluded from the active TB group if they had LTBI codes or were prescribed only one or two anti-TB medications for <4 months.

(2) HZ

Patients with HZ were defined as those who were previously diagnosed with HZ (code B02) (Supplementary Table S1) and redeemed prescription medications for HZ (acyclovir, valacyclovir, or famciclovir) [18]. The diagnostic code for postherpetic neuralgia (code G53.0) was not included. Given the recurrent nature of HZ, a subsequent episode of HZ was considered a new case if it occurred in a different calendar year from the previous one [18].

(3) Malignancy of the bowels

Malignancy of the bowels was defined as a diagnosis of malignant neoplasm of the small (code C17) or large bowels (codes C18–C20), with a V193 RID registration code. After patients with any cancer are registered in the RID system, they become eligible for a 5% co-payment reduction in medical costs for cancer treatment.

(4) Anxiety and Depression

Anxiety and depression are not typically considered direct AEs of ATs in patients with IBD. However, they may occur as part of the disease burden, quality of life or as indirect consequences related to disease control, and there was still insufficient evidence that AT is associated with an increased incidence of anxiety and depression in patients with IBD [19,20]. Therefore, anxiety and depression were included as secondary AEs, and they were defined as a diagnosis of anxiety (codes F40–42) or depression (codes F32–34) (Supplementary Table S1) [11].

### 2.5. Statistical Analysis

Continuous and categorical variables are presented as means ± standard deviation (SD) and number and percentage, respectively. To compare characteristics between the non-AT and AT groups, *t*-tests were used for continuous variables and chi-square tests for binary and categorical variables before and after PSM. Newly developed AEs were estimated using incidence rates (IRs), calculated as the number of event cases divided by the total person-years (PYs) of follow-up. Cox proportional hazards regression was used to estimate the hazard ratios (HRs) for AE occurrence between the AT and non-AT groups. The proportional hazard assumptions were tested for all Cox models. All statistical tests were two-tailed, and the significance level was set at *p* < 0.05. Statistical analyses were performed using R software (version 4.2.3; R Foundation for Statistical Computing, Vienna, Austria).

## 3. Results

A total of 56,335 incident IBD cases (UC = 39,737 and CD = 16,598) were identified after excluding prevalent cases (*n* = 26,007), those without IBD medication use (*n* = 2793), and those with IBD medication used for <90 days (*n* = 6192) (Figure 1). Among patients with IBD, 11,205 patients received ATs and 45,130 patients received non-ATs. In the AT group, 7524 patients were treated with anti-TNF biologics only, 1518 patients with non-anti-TNF biologics only, and 419 with small-molecule agents. A total of 1744 patients who received ATs from more than one class were classified as the other subgroup. The median follow-up duration was 56.0 months (IQR, 62.0).

### 3.1. Baseline Characteristics of Patients with IBD

Table 1 shows the baseline characteristics of patients with IBD according to treatment type before and after PSM. Before PSM, 19.9% (*n* = 11,205) of patients were managed with ATs, while 80.1% (*n* = 45,130) received non-ATs. After PSM, an equal number of patients were matched in a 1:1 ratio for both groups. Before PSM, patients in the AT group were younger, more likely to be male, more frequently diagnosed with CD, more likely to reside in metropolitan areas, and had fewer comorbidities compared with those in the non-AT group. After PSM, however, the AT and non-AT groups were well balanced with respect to mean age, age distribution, sex, type of IBD, location of the medical institution and comorbid diseases. Despite PSM, the AT group had a lower prevalence of DM compared with the non-AT groups.

### 3.2. Active TB Risk in Patients with IBD

The AT group showed a higher IR for TB compared with non-AT group, although the difference was not statistically significant (Table 2). The IR for the non-AT group was 65.4 per 10^5^ PY, based on 31 events over 47,404 PY. The AT group had 60 events over 62,602 PY, yielding an IR of 95.8 per 10^5^ PY, with a HR of 1.2 (95% confidence interval [CI] = 0.8–1.9, *p* = 0.335) relative to non-AT group. When stratified by type of AT, patients receiving anti-TNF biologics had a significantly higher event rate: 100.2 per 10^5^ PY, with an HR of 2.0 (95% CI = 1.4–2.9, *p* < 0.001). In contrast, those receiving non–anti-TNF biologics had an IR of 61.6 with an HR of 1.3 (95% CI = 0.5–3.5, *p* = 0.629), and those on small-molecule agents had an IR of 43.2 with an HR of 0.9 (95% CI = 0.1–6.6, *p* = 0.926), both showing no significant difference from non-AT group.

### 3.3. HZ Risk in Patients with IBD

The IR of HZ for the non-AT group was 2151.3 per 10^5^ PY, based on 706 events over 45,617 PY (Table 3). The AT group had 1256 events over 58,384 PY, corresponding to an IR of 2165.8 per 10^5^ PY. This corresponded to a significantly increased HR of 1.4 compared with conventional therapy (95% CI =1.2–1.5, *p* < 0.001). Within the AT subgroup, patients receiving anti-TNF biologics had an IR of 2092.4 per 10^5^ PY, with an HR of 1.0 (95% CI, 1.0–1.1; *p* = 0.829), indicating no significant difference from non-AT group. Those treated with non–anti-TNF biologics had a lower IR of 1679.0 and a borderline significant reduction in risk (HR = 0.8, 95% CI = 0.7–1.0, *p* = 0.052). In contrast, the small-molecule agent group showed a markedly elevated risk, with an IR of 4134.2 per 10^5^ PY and an HR of 2.0 (95% CI = 1.6–2.5, *p* < 0.001).

### 3.4. Malignancy Risk of the Bowels in Patients with IBD

The IR of non-AT group was 48.5 per 10^5^ PY based on 23 events over 47,385 PYs, while the IR of AT group was 60.8 per 10^5^ PY based on 38 events over 62,544 PY, with no statistically significant difference between two groups (HR = 1.2, 95% CI = 1.0–1.3, *p* = 0.475) (Table 4). Subgroup analysis of AT group showed that patients receiving anti-TNF biologics had an IR of 53.6 per 10^5^ PY, with a non-significant HR of 1.2 (95% CI = 1.1–1.3, *p* = 0.381). Similarly, those receiving non–anti-TNF biologics and small-molecule agents had non-significant HRs (*p* = 0.306 and *p* = 0.700, respectively). However, this analysis was limited by the small number of events and the resulting wide CIs in both groups.

### 3.5. Anxiety or Depression Risk in Patients with IBD

Non-AT group showed 1191 events over 44,159 PY, corresponding to an IR of 2697.1 per 10^5^ PY (Table 5). In the AT group, 1472 events occurred over 56,897 PY, yielding an IR of 2587.1 per 10^5^ PY, with no statistically significant difference compared with non-AT group (HR = 0.9, 95% CI = 0.9–1.1, *p* = 0.499). In subgroup analyses, patients treated with anti-TNF biologics had an IR of 2517.9 per 10^5^ PY and a significantly reduced risk compared with non-AT group (HR = 0.8, 95% CI = 0.8–0.9, *p* < 0.001). Similarly, those receiving non–anti-TNF biologics showed a lower IR of 2344.0 and a significantly lower hazard (HR = 0.8, 95% CI = 0.7–0.9, *p* = 0.004). In contrast, patients using small-molecule agents had a higher IR of 3499.2, though the increase in risk was not statistically significant (HR = 1.2, 95% CI = 0.9–1.5, *p* = 0.167).

### 3.6. ER Hospitalization, Surgery and Death in Patients with IBD

Medical costs, ER hospitalizations, surgical interventions and mortality were compared between AT group and non-AT group before and after PSM (Supplementary Table S2). After PSM, AT group showed significantly higher mean medical costs, more frequent ER hospitalizations with higher number of hospitalizations (all *p* < 0.001). However, the cumulative number of hospitalization days was lower in the AT group (*p* < 0.001). Surgery rates were also significantly higher in the AT group (*p* < 0.001), whereas mortality did not differ significantly between the two groups after matching (*p* = 0.082).

## 4. Discussion

Only a few population-based studies have evaluated AEs associated with IBD medications. The major clinical implication of this study lies in its use of a nationwide, propensity score-matched cohort to compare AEs between patients receiving ATs, and those on non-AT regimens.

The increased risk of active TB with anti-TNF therapy is well recognized, especially in Asian countries [21]. Recent studies from Western countries have reported relatively low TB incidence during anti-TNF therapy [22,23,24]. In a US registry of 6273 patients with CD, TB incidence was only 0.01 per 100 person-years and did not differ between the infliximab and conventional therapy [22]. Similarly, a French cohort study reported a modest increase in TB risk with anti-TNF monotherapy compared to thiopurine monotherapy (HR = 2.0, 95% CI = 1.2–3.4) [24]. In contrast, the risk of active TB associated with anti-TNF therapy is much higher in Asian countries [21]. A nationwide population-based study from Korea revealed a 223.9/10^5^ PYs incidence rate of active TB during anti-TNF therapy for IBD treatment [5]. In addition, the incidence rate of TB was 1.9–3.9 times higher with anti-TNF therapy than with conventional therapy, which is comparable to our findings. This previous Korean study differed from the present study because TB risk was analyzed between patients with UC and CD, between patients with IBD and the general population, and between anti-TNF therapy (only infliximab and adalimumab) and non-ATs. The risk of TB may be higher than our findings because the previous Korean study was based on data from 2011 to 2013, when TB screening and prophylaxis were less commonly performed due to limited awareness in Korea. Our study showed that only anti-TNF therapy was associated with an increased risk of active TB compared with conventional therapy, with a rate of 100.2/10^5^ PYs (HR = 1.9, 95% CI = 1.4–2.7, *p* < 0.001). In contrast, non-anti-TNF biologics (such as vedolizumab and ustekinumab) were not associated with an increased risk of TB compared with conventional therapy, which is consistent with the safety profiles of non-anti-TNF biologics [23]. The lack of an association between small-molecule agent (tofacitinib) and TB may be explained by the small number of patients receiving tofacitinib, which was approved in Korea in 2019. In a study based on 19,406 PYs in patients with rheumatoid arthritis, tofacitinib was associated with slightly increased risk of TB, with an incidence rate of 0.2/100 PYs [25]. Therefore, the risk of TB associated with small-molecule agents should be re-evaluated with large-scale data in patients with IBD.

Whether anti-TNF therapy is associated with HZ risk is unclear, because the mechanism by which anti-TNF therapy causes HZ has not been elucidated. In a large cohort study, the crude incidence rate of HZ among anti-TNF users was 11.3/1000 PYs in patients with IBD; however, no significant difference in HZ rates was observed between anti-TNF therapy and conventional therapy after PSM [24]. In the present study, anti-TNF therapy and non-anti-TNF biologics were not associated with an increased HZ incidence rate compared with conventional therapy. However, small-molecule agent (tofacitinib) was associated with an increased risk of HZ, with a rate of 4134.2/10^5^ PYs (HR = 2.0, 95% CI = 1.6–2.5, *p* < 0.001), which was consistent with previous reports [23,26,27]. Multiple studies have demonstrated an increased risk of HZ with tofacitinib treatment, and the pooled incidence rate of HZ is 2.67/100 PYs [27]. In addition, the incidence rate of HZ was higher in Asian populations, including Koreans, with a rate of 4.4/100 PYs among patients receiving tofacitinib for rheumatoid arthritis [28]. Given the markedly elevated risk of HZ observed in the tofacitinib group, particularly among Asian patients, pre-treatment screening and proactive vaccination strategies should be strongly recommended as part of routine clinical care. However, the generalizability of our recommendations to non-Asian populations may be limited as the epidemiology of TB and HZ varies substantially across geographic regions.

In addition, the AT cohorts in our study largely reflect patients who had already been exposed to corticosteroids and immunomodulators before the initiation of AT. Consequently, the observed safety outcomes in our study should be interpreted within a treatment paradigm, in which ATs are introduced after conventional immunosuppressive therapy, which is especially important for TB and HZ risk.

The risk of bowel malignancies associated with ATs in patients with IBD should not be overlooked. For example, lymphoma, non-melanoma skin cancer, and cervical cancer have been reported with anti-TNF therapy, and lung cancer and lymphoma have been reported with tofacitinib use [23]. Compared with conventional therapy, we found no association between AT and the risk of bowel malignancy. However, these findings should be interpreted with caution, as they are limited by the small number of events across all groups and by the focus solely on bowel malignancies. Given the long latency required for malignancy development, furthermore, the follow-up duration and exposure timing in this study may be insufficient to fully assess malignancy risk. Therefore, longer-term prospective studies with larger patient populations are needed to better assess the risk of malignancies.

IBD is a lifelong disease without a cure; consequently, the prevalence of anxiety or depression in patients with IBD is high [29]. In addition, patients with IBD and anxiety or depression are at an increased risk of relapse, hospitalization, emergency department visits, and surgery [30]. Recent consensus statement on managing anxiety and depression in patients with IBD emphasized healthcare provider awareness of these conditions [19]. A systematic review reported a risk difference of only 0.01 adverse psychiatric events per 100 person-months with biologic therapies (95% CI = 0.00–0.02) [20]. However, that study differed from ours in that it included all psychiatric outcomes—such as suicide, suicidal ideation, psychosis, in addition to anxiety and depression—and was limited by heavy reliance on longitudinal, observational studies. From this perspective, our findings that anxiety or depression were less likely to occur in patients being treated with anti-TNF biologics (HR = 0.8, 95% CI = 0.8–0.9, *p* < 0.001) and non-anti-TNF biologics (HR = 0.8, 95% CI = 0.7–0.9, *p* = 0.004) compared with those receiving conventional therapy may address this knowledge gap. Low IR of anxiety and depression may be explained by the alleviation of IBD symptoms or the reduced risk of relapse with these medications compared with conventional therapy. However, the association between ATs and anxiety/depression should be cautiously reevaluated from well-designed, larger studies on this issue.

After PSM, the AT group demonstrated significantly higher overall medical costs and a higher frequency of hospitalizations, compared with the non-AT group. These findings likely reflect the complexity of disease management in patients receiving ATs, who may have more severe disease or require closer surveillance. The significantly higher healthcare costs and surgery rates in this group highlight the importance of early risk stratification and optimal therapeutic selection to balance treatment efficacy with resource burden. These results underscore the need to consider both clinical outcomes and healthcare resource utilization in evaluating the real-world impact of ATs in IBD management.

The safety of biosimilars should be interpreted in the context of the existing literature on pharmacovigilance [31,32]. Although biosimilars undergo a rigorous comparability pathway and are expected to have no clinically meaningful differences from their reference products, they are not identical, and long-term safety profiles should not be assumed to be fully interchangeable. Given the inherent complexity of biologics, safety may be influenced by immunogenicity, manufacturing variability, and limitations in traceability. Accordingly, sustained post-marketing surveillance remains essential to detect rare AEs, switching-related outcomes, and product-specific safety signals in routine clinical practice. In this context, we intended to perform a subgroup analysis comparing AEs between biosimilars and originator biologics. However, due to the structure of the database and restrictions related to data access, AT medications could only be analyzed at the group level (e.g., anti–TNF biologics) rather than by individual drug names. Accordingly, it was not feasible to separately evaluate AEs specific to biosimilars in the present study.

The present study had certain limitations. First, a nationwide claims database with comprehensive population coverage was used in our study; however, generalizability may be limited due to regional differences in AE prevalence. Nevertheless, our large-scale analysis provides valuable insights for Asian populations. Second, the retrospective design precludes causal inference, although propensity matching minimized confounding. However, retrospective real-world data may provide important safety information that is not fully captured in clinical trials. Third, we used Cox proportional hazards models based on time-to-event data and calculated IRs per PYs, which account for differences in follow-up time and allow estimation of risk over the observation period. However, follow-up duration and cumulative drug exposure were not directly considered. In addition, differences in follow-up initiation between the AT and non-AT groups may introduce immortal time bias that cannot be fully mitigated by excluding pre-treatment events or by applying Cox models. Accordingly, this study design inherently limits the ability to achieve a fully unbiased comparison between groups, and the findings should be interpreted with caution. Fourth, our database was not originally constructed as an IBD-specific clinical cohort and lacks detailed information on key clinical confounders, including disease activity and treatment history. Although under the Korean NHIS reimbursement criteria, ATs for IBD are generally restricted to patients with moderate-to-severe disease who have failed or are intolerant to conventional therapies, this proxy does not fully capture disease severity at the individual level. Given the well-established association between disease severity and AE risk, our analysis is therefore subject to substantial residual confounding, which cannot be fully addressed with the available data. Accordingly, our findings should be interpreted with caution, and no causal inferences should be drawn from the comparative risk estimates. Fifth, we performed 1:1 PSM using only the two most critical variables, age and IBD type; however, we acknowledge that additional potential confounding covariates—such as sex, comorbidities, prior hospitalization or surgery, steroid or immunomodulator use, healthcare utilization, and calendar year—may also influence comparative safety outcomes. While an extensive PSM incorporating these factors would be ideal, our matching was necessarily limited to age and IBD type due to the relatively low number of events in the AT and non-AT groups, which could render meaningful comparisons challenging, particularly for AT subgroups. Sixth, we did not account for combination therapy use in the anti-TNF group. Given that combination regimens are common in Korea, our results likely reflect combination therapy outcomes. Finally, Data granularity was limited by claims-based definitions. However, the RID registry ensures high diagnostic accuracy for IBD and its treatment patterns. AE definitions also relied on diagnostic codes, though the Korea NHIS offers good coding fidelity for IBD-related outcomes.

## 5. Conclusions

In summary, this nationwide population-based study found that overall AT use was not associated with an increased risk of TB, whereas anti–TNF therapy was associated with a higher risk of TB. AT use was also associated with an increased risk of HZ, particularly with small-molecule agents, and with a lower risk of anxiety/depression. Beyond safety profiles, the markedly increased healthcare utilization in AT group highlights the importance of aligning clinical benefit with healthcare sustainability. These findings emphasize the need for individualized, risk-benefit-based decision-making to ensure optimal and efficient IBD management.

## Figures and Tables

**Table 1:** Baseline characteristics of patients with IBD according to treatment type before and after propensity score matching.

Demographics	Before Propensity Score Matching			After Propensity Score Matching		
	AT Group (*n* = 11,205)	Non-AT Group (*n* = 45,130)	*p* Value	AT Group (*n* = 11,205)	Non-AT Group (*n* = 11,205)	*p* Value
Age (year), mean (SD)	31.0 (14.8)	40.6 (16.3)	<0.001	31.0 (14.8)	31.2 (14.7)	0.318
Age groups, *n* (%)			<0.001			1.000
17–40 years	8444 (75.4)	23,159 (51.3)		8444 (75.4)	8444 (75.4)	
41–64 years	2335 (20.8)	17,927 (39.7)		2335 (20.8)	2335 (20.8)	
≥65 years	426 (3.8)	4044 (9.0)		426 (3.8)	426 (3.8)	
Sex (male), *n* (%)	7838 (70.0)	28,804 (63.8)	<0.001	7838 (70.0)	7957 (71.0)	0.084
Type of IBD, *n* (%)			<0.001			1.000
Crohn’s disease	6758 (60.3)	9840 (21.8)		6758 (60.3)	6758 (60.3)	
Ulcerative colitis	4447(39.7)	35,290 (78.2)		4447 (39.7)	4447 (39.7)	
Location of medical institution, *n* (%)			<0.001			0.324
Metropolitan area	4351 (38.8)	16,724 (37.1)		4351 (38.8)	4424 (39.5)	0.324
Non-metropolitan area	6854 (61.2)	28,406 (62.9)		6854 (61.2)	6781 (60.5)	
Comorbid disease, *n* (%)						
Diabetes mellitus	1456 (13.0)	8519 (18.9)	<0.001	1456 (13.0)	1276 (11.4)	0.004
Hypertension	1749 (15.6)	11,402 (25.3)	<0.001	1749 (15.6)	1624 (14.5)	0.132
Obesity	69 (0.6)	235 (0.5)	0.217	69 (0.6)	58 (0.51)	0.535
Chronic pulmonary disease	7749 (69.2)	31,160 (69.0)	0.258	7749 (69.2)	7507 (66.9)	0.254
Renal disease	262 (2.3)	1127 (2.5)	0.439	262 (2.3)	209 (1.9)	0.059
Cardiovascular disease	761 (6.8)	4813 (10.7)	<0.001	761 (6.8)	696 (6.2)	0.217
Any malignancy	436 (3.9)	2948 (6.5)	<0.001	436 (3.9)	443 (4.0)	0.361

Abbreviations: AT, advanced therapy; SD, standard deviation; IBD, inflammatory bowel disease.

**Table 2:** Occurrence of tuberculosis in patients with IBD according to treatment type after propensity score matching.

Treatment Type	Event Case	Person Years	IR (per 10^5^ Person-Years) (95% CI)	HR (95% CI)	*p* Value
Conventional therapy (Ref.)	31	47,404	65.4 (95% CI, 42.2–88.6)	Ref.	
Advanced therapy	60	62,602	95.8 (95% CI, 72.0–120.0)	1.2 (95% CI, 0.8–1.9)	0.335
Subgroup of AT					
Anti-TNF biologics	43	42,932	100.2 (95% CI, 71.0–130.0)	2.0 (95% CI, 1.4–2.9)	<0.001
Non-anti-TNF biologics	4	6496	61.6 (95% CI, 1.30–122.0)	1.3 (95% CI, 0.5–3.5)	0.629
Small-molecule agent	1	2316	43.2 (95% CI, 18.0–104.0)	0.9 (95% CI, 0.1–6.6)	0.926

Abbreviations: IR, incidence rate; PY, person year; CI, confidence interval; HR, hazard ratio; AT, advanced therapy.

**Table 3:** Risk and incidence rates of herpes zoster in patients with IBD according to treatment type after propensity score matching.

Risk	Event Case	Person Years	IR (per 10^5^ Person-Years) (95% CI)	HR (95% CI)	*p* Value
Conventional therapy (Ref.)	706	45,617	2151.3 (95% CI, 1435.0–1661.0)	Ref.	
Advanced therapy	1256	58,384	2165.8 (95% CI, 2047.6–2283.8)	1.4 (95% CI, 1.2–1.5)	<0.001
Subgroup of AT					
Anti-TNF biologics	840	40,145	2092.4 (95% CI, 1952.4–2232.4)	1.0 (95% CI, 1.0–1.1)	0.829
Non-anti-TNF biologics	104	6194	1679.0 (95% CI, 1359.1–1999.0)	0.8 (95% CI, 0.7–1.0)	0.052
Small-molecule agent	85	2056	4134.2 (95% CI, 3273.6–4994.7)	2.0 (95% CI, 1.6–2.5)	<0.001

Abbreviations: IR, incidence rate; PY, person year; CI, confidence interval; HR, hazard ratio; AT, advanced therapy.

**Table 4:** Occurrence of malignancy in patients with IBD according to treatment type after propensity score matching.

Risk	Event Case	Person Years	IR (per 10^5^ Person-Years) (95% CI)	HR (95% CI)	*p* Value
Conventional therapy (Ref.)	23	47,385	48.5 (95% CI, 28.6–68.3)	Ref.	
Advanced therapy	38	62,544	60.8 (95% CI, 41.4–80.0)	1.2 (95% CI, 1.0–1.3)	0.475
Subgroup of AT					
Anti-TNF biologics	23	42,901	53.6 (95% CI, 31.7–75.5)	1.2 (95% CI, 1.1–1.3)	0.381
Non-anti-TNF biologics	2	6495	30.8 (95% CI, 0.0–73.4)	0.8 (95% CI, 0.7–1.0)	0.306
Small-molecule agent	1	2317	43.2 (95% CI, 0.0–127.7)	1.6 (95% CI, 1.2–2.0)	0.700

Abbreviations: IR, incidence rate; PY, person year; CI, confidence interval; HR, hazard ratio; AT, advanced therapy.

**Table 5:** Occurrence of anxiety/depression in patients with IBD according to treatment type after propensity score matching.

Risk	Event Case	Person Years	IR (per 10^5^ Person-Years) (95% CI)	HR (95% CI)	*p* Value
Conventional therapy (Ref.)	1191	44,159	2697.1 (95% CI, 2545.3–2848.1)	Ref.	
Advanced therapy	1472	56,897	2587.1 (95% CI, 2456.7–2717.5)	0.9 (95% CI, 0.9–1.1)	0.499
Subgroup of AT					
Anti-TNF biologics	985	39,119	2517.9 (95% CI, 2362.7–2673.2)	0.8 (95% CI, 0.8–0.9)	<0.001
Non-anti-TNF biologics	142	6058	2344.0 (95% CI, 1963.0–2725.0)	0.8 (95% CI, 0.7–0.9)	0.004
Small-molecule agent	71	2029	3499.2 (95% CI, 2699.6–4298.8)	1.2 (95% CI, 0.9–1.5)	0.167

Abbreviations: IR, incidence rate; PY, person year; CI, confidence interval; HR, hazard ratio; AT, advanced therapy.

## Data Availability

The data presented in this study are available on reasonable request from the corresponding author. The data are not publicly available due to privacy and legal restrictions.

## Supplementary Materials

The following supporting information can be downloaded at: https://www.mdpi.com/article/10.3390/jcm15072562/s1, Table S1. ICD-10 codes used in this study. Table S2. Medical cost, hospitalization, surgical intervention and mortality in patients with IBD before and after propensity score matching.
