# Supplementary material for: Adverse Events in Patients with Inflammatory Bowel Disease Treated with Advanced Therapies: A Nationwide, Population-Based, Propensity-Matched Cohort Study
Source: J Clin Med. 2026 Mar 27;15(7):2562. doi: 10.3390/jcm15072562 (PMC13073948; doi:10.3390/jcm15072562)
Supplement: Supplementary file 1 [file jcm-15-02562-s001.zip › jcm-4181308-supplementary.pdf]

Supplementary Table S1. ICD-10 codes used in this study.

Variables	ICD-10 codes
Comorbidities	
Diabetes mellitus	E11: type 2 diabetes mellitus; E12: malnutrition-related diabetes mellitus; E13: other specified diabetes mellitus; E14: unspecified diabetes mellitus
Hypertension	I10: essential (primary) hypertension; I11: hypertensive heart disease; I12: hypertensive chronic kidney disease; I13: hypertensive heart and chronic kidney disease; I15: secondary hypertension
Congestive heart failure	I50: heart failure
Ischemic heart disease	I20: angina pectoris; I21: acute myocardial infarction; I22: subsequent myocardial infarction; I23: certain current complications following acute myocardial infarction; I24: other acute ischemic heart diseases; I25: chronic ischemic heart disease
Cerebrovascular disease	I63: cerebral infarction; I64: stroke, not specified as hemorrhage or infarction
Chronic obstructive pulmonary disease	J41: simple and mucopurulent chronic bronchitis; J42: unspecified chronic bronchitis; J43: emphysema; J44: other chronic obstructive pulmonary disease
Renal disease	N18: chronic kidney disease; N19: unspecified kidney failure; Z49: encounter for care involving renal dialysis; Z94.0: kidney transplant status; Z99.2: dependence on renal dialysis
Small and large bowel surgery	Q2650, Q2651: resection of small intestine; Q2671: right or left hemicolectomy; Q2672: total colectomy; Q2679: Hartmann's procedure; Q2680: intestinal anastomosis; Q2691: surgery for intestinal obstruction including resection of intestine; Q2921: anterior resection; Q2922: low anterior resection; Q2923: abdominoperineal resection (the Miles operation); Q2924: abdominal pull through surgery; Q2925, Q2926: total coloprotectomy
Adverse events	

TB	A15: respiratory TB, bacteriologically and histologically confirmed; A16: respiratory TB, bacteriologically or histologically not confirmed; A17: TB of the nervous system; A18: TB of other organs; A19: miliary TB; U88: multidrug-resistant tuberculosis (MDR-TB)
Herpes zoster	B02: zoster (B02.0: zoster encephalitis; B02.1: zoster meningitis; B02.2: zoster with other nervous system involvement; B02.3: zoster ocular disease; B02.7: disseminated zoster; B02.8: zoster with other complications; B02.9: zoster without complications)
Anxiety	F40: phobic anxiety disorder; F41: other anxiety disorders; F42: obsessive-compulsive disorder
Depression	F32: major depressive disorder, single episode; F33: major depressive disorder, recurrent; F34: persistent mood-affective disorders
Malignancy	C17: malignant neoplasm of the small intestine; C18: malignant neoplasm of the colon; C19: malignant neoplasm of the rectosigmoid junction; C20: malignant neoplasm of the rectum

Abbreviations: ICD-10, International Classification of Diseases, 10th revision; TB, tuberculosis

Supplementary Table S2. Medical cost, hospitalization, surgical intervention and mortality in patients with IBD before and after propensity score matching.

	Before propensity score matching			After propensity score matching		
**Variables**	**AT group (n=11,205)**	**Non-AT group (n=45,130)**	***P* value**	**AT group (n=11,205)**	**Non-AT group (n=11,205)**	***P* value**
Medical cost, (×10^5 KRW), mean (SD)	10.7 (20.3)	3.6 (11.6)	<0.001	10.7 (20.3)	4.8 (14.2)	<0.001
Hospitalization via ER, n (%)	6582 (58.7)	15,500 (34.4)	<0.001	6582 (58.7)	4552 (40.6)	<0.001
Number of hospitalizations, mean (SD)	6 (7)	4 (4)	<0.001	6 (7)	4 (5)	<0.001
Cumulative days, median (IQR)	438 (486)	606 (712)	<0.001	438 (486)	617 (691)	<0.001
Surgery, n (%)	1226 (10.9)	1086 (2.4)	< 0.001	1226 (10.9)	603 (5.4)	<0.001
Death, n (%)	92 (0.8)	487 (1.1)	0.018	92 (0.8)	69 (0.6)	0.082

Abbreviations: SD, standard deviation; ER, emergency room; IQR, interquartile range
